# Stromal Activation by Tumor Cells: An *in Vitro* Study in Breast Cancer

**DOI:** 10.3390/microarrays5020010

**Published:** 2016-05-18

**Authors:** Giuseppe Merlino, Patrizia Miodini, Biagio Paolini, Maria Luisa Carcangiu, Massimiliano Gennaro, Matteo Dugo, Maria Grazia Daidone, Vera Cappelletti

**Affiliations:** 1 Department of Experimental Oncology and Molecular Medicine, Fondazione IRCCS Istituto Nazionale dei Tumori, Via Amadeo 42, 20133 Milan , Italy; peppem1985@gmail.com (G.M.); patrizia.miodini@istitutotumori.mi.it (P.M.); mariagrazia.daidone@istitutotumori.mi.it (M.G.D.); 2 Department of Pathology, Fondazione IRCCS Istituto Nazionale dei Tumori, Via Venezian 1, 20133 Milan , Italy; biagio.paolini@istitutotumori.mi.it (B.P.); marialuisa.carcangiu@istitutotumori.mi.it (M.L.C.); 3 Department of Surgery, Fondazione IRCCS Istituto Nazionale dei Tumori, Via Venezian 1, 20133 Milan , Italy; massimiliano.gennaro@istitutotumori.mi.it; 4 Functional Genomics Core Facility, Department of Experimental Oncology and Molecular Medicine, Fondazione IRCCS Istituto Nazionale dei Tumori, Via Amadeo 42, 20133Milan, Italy; matteo.dugo@istitutotumori.mi.it

**Keywords:** breast cancer, stroma-tumor interaction, stromal activation, fibroblasts, biomarkers, ductal carcinoma *in situ*, *in-vitro* models, gene expression profiles

## Abstract

Background: The tumor microenvironment participates in the regulation of tumor progression and influences treatment sensitivity. In breast cancer, it also may play a role in determining the fate of non-invasive lesions such as ductal carcinoma *in situ* (DCIS), a non-obligate precursor of invasive diseases, which is aggressively treated despite its indolent nature in many patients since no biomarkers are available to predict the progression of DCIS to invasive disease. *In vitro* models of stromal activation by breast tumor cells might provide clues as to specific stromal genes crucial for the transition from DCIS to invasive disease. Methods: normal human dermal fibroblasts (NHDF) were treated under serum-free conditions with cell culture media conditioned by breast cancer cell lines (SkBr3, MDA-MB-468, T47D) for 72 h and subjected to gene expression profiling with Illumina platform. Results: *TGM2*, coding for a tissue transglutaminase, was identified as candidate gene for stromal activation. In public transcriptomic datasets of invasive breast tumors *TGM2* expression proved to provide prognostic information. Conversely, its role as an early biosensor of tumor invasiveness needs to be further investigated by *in situ* analyses. Conclusion: Stromal *TGM2* might probably be associated with precancerous evolution at earlier stages compared to DCIS.

## 1. Introduction

Tumors arise within a microenvironment, which cannot be considered a simple bystander since it plays an active role in the acquisition of the malignant traits of the diseases to such an extent, that it may even be regarded as a therapeutic target [1,2,3,4,5,6]. The tumor microenvironment consists of the extracellular matrix (ECM) and various cells of hematopoietic and mesenchymal origin, such as cells from the lymphoid lineage (T cells, B cells and natural killer (NK) cells) or the myeloid lineage such as macrophages, neutrophils and myeloid-derived suppressor cells. Mesenchymal cells such as fibroblasts, myofibroblasts and adipocytes also share the microenvironment with the tumor cells. Current molecular subtyping and prognostic assessment of breast tumors is based on molecular features of cancer cells, but numerous stromal signatures with clinical validity have also been defined [7,8].

The current paradigm indeed envisages a strict connection between evolution of epithelial and stromal compartments, which is resulting into a symbiotic relation when tumor cells co-opt stromal cells to activate tumor promoting programs such as acquirement of invasive traits [9,10], or in a restrictive interaction when the microenvironment offers a barrier to tumor development [11]. The latter is particularly evident considering the frequently observed local immunological response mediated by infiltrating immune and inflammatory cells. Infiltrating lymphocytes and lately immune signatures do indeed represent tools predicting better prognosis and major sensitivity to chemotherapy especially in triple negative (TNBC) and in Her2 breast tumor [12,13].

Although they are the result of a co-evolution with the tumor, changes in the stromal compartment occurring in response to the development and progression of a tumor share to a certain extent traits that are common among different tumor types. Common and distinct mechanisms shared by activated fibroblasts have been recently reviewed in different malignant contexts such as breast, prostate and lung tumors [14,15]. Cancer associated fibroblasts (CAFs), although still eluding a clear-cut definition, often represent the most abundant cells in the breast tumor microenvironment, and behave as activated fibroblasts producing ECM components such as collagens, proteoglycans, cytokines, proteases and growth factors [15].

In breast cancer, DCIS has been recognized as a non-obligate precursor of invasive disease. In fact, a variable proportion (25%–50%, mainly depending on histological grade) of patients diagnosed with DCIS progress to invasive disease. This raises many unanswered questions as to the biological reasons for progression and constitutes a clinical dilemma for the management of DCIS patients that consequently often undergo over-treatment [16]. Invasive ipsilateral recurrence are the result of host- and tumor-specific factors, and tools such as the early developed Van Nuys prognostic index [17] or other later developed nomograms [18] are trying to define accurate predictions. Nonetheless, the field is still awaiting biologically-derived biomarkers able to refine prediction.

In this study, we hypothesized that studying *in vitro* the transcriptional activation of normal fibroblasts in the presence of a tumor might inform on tumor-subtype specific alterations occurring in the stromal compartment of clinical tumors offering hints on biomarkers reflecting the early sensing of the stroma in the presence of the incipient tumor. We therefore developed an *in vitro* approach to model the early activation of fibroblasts. Our approach was focused on paracrine-mediated signals as it included the treatment of normal, dermal-derived fibroblast with the secretome (tumor conditioned medium) obtained from three distinct breast cancer cell lines, representing the luminal, the HER2-enriched and the triple negative molecular subtypes. Genes showing perturbations at the transcriptional level were considered as possible candidate biomarkers recapitulating an early activation of the stromal compartment. The hypothesis was challenged in the context of clinical DCIS. We identified *TGM2* as a possible stromal early sensor of the malignancy and its role should be tested in earlier stages of malignancy development.

## 2. Material and Methods

### 2.1. Cell Lines

Human breast cell lines (BCCLs), SkBr3, MDA-MB-468, T47D, purchased from the American Type Culture Collection, were cultured in McCoy’s 5A (SkBr3) or Dulbecco’s modified Eagle’s medium (MDA-MB-468, T47D) (Lonza, Slough, UK) supplemented with 5% (T47D) or 10% fetal bovine serum (Lonza). The human normal fibroblast (NF) cell line NHDF (normal human dermal fibroblasts), derived from human normal derma (Lonza) was cultured in Fibroblast Basal Medium (FBM) supplemented with Fibroblast Growth Medium-2 (FGM-2) Bullet kit (Lonza), containing 2% fetal bovine serum (FBS), 0.1% Insulin, 0.1% gentamicin, amphotericin GA 1000, 0.1% fibroblast growth factor (FGF). All cell lines were cultured at 37 °C in 95% humidified air in the presence of 5% CO_2_ and authenticated with short tandem repeat DNA profiling analysis by the Functional Genomic Unit of the Department of Experimental Oncology at Fondazione IRCCS Istituto Nazionale Tumori of Milano (INT).

### 2.2. Conditioned Medium Collection

Conditioned media (c.m.) were collected from BCCLs (SkBr3, MDA-MB-468, T47D) separately plated (1.65 × 10^6^ cells) in F25 cm^2^ flasks, in DMEM F/12 5% FBS/FBM 2% FBS (1:1) thereafter referred as MIX medium. After cell attachment, the medium was replaced with 7 mL of serum-free MIX medium and collected at 72 h. After collection, media were clarified by centrifugation (1400× *g* for 3 min).

C.m. produced by SkBr3, MDA-MB-468, T47D were used for the treatment (72 h) of NHDF (NAF) plated in 6-wells plate at a density of 750,000 cells.

### 2.3. Flow Cytometry Analysis

Fibroblasts cell suspension was washed and incubated in staining solution containing bovine serum albumin (BSA) 1% and EDTA 2 mM with specific antibodies used at appropriate dilution as indicated by datasheet. The following antibodies, directed against extracellular antigens, were employed: FITC anti-human CD90 (cat.# 11-0909-41, eBiosciences, Hatfield, UK) at a 1:50 dilution, PE anti-human CD105 (cat.# FAB10971P, R&D Systems Inc, Minneapolis, MB, USA) at a 1:10 dilution, PE anti-human CD166 (cat.#559263, BD Biosciences, San Jose, CA, USA ) at a 1:10 dilution CD73 (cat.#550257, BD Biosciences) at a 1:10 dilution. For detection of the intracellular expression of α- Smooth Muscle Actin, cells were fixed with paraformaldehyde 4% and permeabilized with a solution containing 0.5% saponin, 0.1% BSA in phosphate buffered saline. The anti-human α-Smooth Muscle Actin antibody (R&D Systems, Minneapolis, MN, USA) was used at 1:50 dilution in the permeabilization solution.

### 2.4. RNA Extraction and Microarray Hybridization

Total RNA was extracted from treated NHDF cells using Qiazol (Qiagen, Valencia, CA, USA) reagent followed by a clean-up treatment with the RNeasy Mini kit (Qiagen) according to manufacturer’s recommendations to remove contaminating genomic DNA. RNA integrity and purity were assessed by Bioanalyzer (Agilent Technologies, Waldbronn, Germany) and concentration was evaluated using a NanoDrop 2000c spectrophotometer (Thermo Scientific, Waltham, MA, USA).

RNA samples were processed for microarray hybridization by INT Functional Genomics core facility. Briefly, 300 ng of total RNA were reverse-transcribed, labeled with biotin and amplified overnight with the Illumina RNA TotalPrep Amplification kit (Ambion, Austin, TX, USA) according to manufacturer’s instructions. One µg of biotinylated cRNA was mixed with the Hyb E1 hybridizatioin buffer containing 37.5% (*w*/*w*) formamide and then hybridized to Illumina HumanHT-12v4 Expression BeadChip (Illumina, Inc., San Diego, CA, USA) at 58 °C for 18 h. Arrays were washed with manufacturer’s E1BC solution (Illumina Inc, San Diego, CA, USA), stained with 1 µg/mL Cy3-streptavidine (GE Healthcare, Buckinghamshire, UK) and scanned with Illumina BeadArray Reader. Illumina BeadScan software was used for image acquisition and recovery of primary signals.

### 2.5. qPCR

Expression levels for *TGM2*, *IL6* and *TGFB*, were evaluated by qPCR with TaqMan Fast Universal PCR Master Mix assay (Thermo Scientific) using *GAPDH* as housekeeping gene. Following primers were obtained from Applied Biosystems (Foster City, CA, USA): *TGM2*, assay Hs00190278_m1; *IL6* assay Hs00985639_m1; *TGFB*, assay Hs00998133_m1; *GAPDH*, Hs00266705_g1). cDNA was generated from 400 ng of RNA. Reverse transcription was run for 10 min at 25 °C, followed by 60 min at 42 °C and 5 min at 85 °C with the High-Capacity cDNA Reverse Transcription kit (Thermo Scientific) in a total volume of 20 µL, according to the manufacturer’s instructions. Data were computed with the ΔΔ*C*t method [19].

### 2.6. Protein Studies

For evaluation of secreted TGM2 protein, 7.5 × 10^5^ NHDFs were seeded on 6-wells and grown for 72 h with BCCL-derived c.m. or control medium. At the end of the treatment, dishes were washed twice with PBS and treated with a hypotonic buffer (NH_4_OH 20 mM) for 20 min. After two more washes with PBS, extracellular matrix proteins deposed on the plastic dish were directly recovered in loading buffer with the help of a scraper and analyzed for TG2 presence by Western blotting using the mouse monoclonal anti-TG2 antibody (CUB 7402, cat.#ab2386, Abcam, Cambridge, UK). TGM2 protein levels in cell-derived extracellular matrix were normalized respect to the number of seeded cells grown in parallel to the cells used for extracellular matrix recovering.

For evaluation of intracellular TGM2 protein, cells were lysed in Laemmli sample buffer containing 5% β-mercaptoethanol and boiled for 3 min. Aliquots containing 80 µg of total cell proteins were fractionated on 12% sodium dodecyl sulfate-polyacrylamide gel electrophoresis (SDS-PAGE) and transferred to nitrocellulose membranes. Membranes were blocked in 3% nonfat milk in Tris-buffered saline for 20 min at room temperature and then incubated overnight at 4 °C with mouse monoclonal anti-TG2 antibody. After washing in Tris-buffered saline containing 0.1% Tween 20, the filters were incubated with peroxidase anti-rabbit immunoglobulin G, and specific complexes were revealed by chemiluminescence according to the Amersham enhanced chemiluminescence Western blotting Detection kit (GE Healthcare, Buckinghamshire, UK). TGM2 protein levels were densitometrically analyzed and normalized for ß-actin protein expression evaluated on the same gel.

### 2.7. Data Analysis and Statistics

#### 2.7.1. Microarray Data Analysis

The Illumina BeadStudio software version 3.8 was used to retrieve microarray raw data and the lumi [20] Bioconductor package was employed for data pre-processing. After quality control, robust spline normalization was applied to log2-transformed data and probes with a detection *p*-value < 0.01 in at least one sample were selected. For genes represented by multiple probes we selected the probe detected in the highest number of samples (according to detection *p*-value < 0.01). In case of ties, the probe with the highest interquartile range was chosen. Raw and processed data were deposited to the Gene Expression Omnibus data repository [21] with accession number GSE80035. The limma [22] Bioconductor package (R version 2.15.2) was used for differential expression analysis. The Benjamini-Hochberg false discovery rate (FDR) method was applied to adjust for multiple testing. Genes with an FDR < 0.0001 and a log2 fold change < −1 or > 1 were selected as differentially expressed (DE). Gene Set Enrichment Analysis (GSEA) [23] was run in pre-ranked mode using pre-ranked gene lists according to the t statistic obtained from differential expression analysis with limma. A total of 1843 gene sets, including canonical pathways (c2) and gene ontology (c5) collections from MSigDB database (http://software.broadinstitute.org/gsea/msigdb) were tested. Enrichment was considered significant at FDR < 0.05.

#### 2.7.2. Statistical and Survival Analyses

Differences in two-group comparisons of continuous variables were assessed using two-tailed unpaired or paired Student’s *t*-test, as appropriate. For comparisons involving more than two groups we applied ANOVA followed by Tukey’s post-*hoc* test.

Association between TGM2 expressions with outcome was evaluated using the Kaplan-Meier method. Samples included in each dataset were classified in TGM2-high or TGM2-low according to whether the expression of *TGM2* was greater than its median expression. Disease-specific survival (DSS) was the main end point in the METABRIC cohort while distant metastasis free survival (DMFS) was the endpoint in the combined dataset. Survival differences were evaluated using the log-rank test. All observations were censored at 10 years of follow-up. A *p*-value < 0.05 was considered for statistical significance.

### 2.8. Case Series

Samples collected at our Institution were used for immunohistochemical detection of TG2. Ten patients with histologically confirmed DCIS, median age 60 years (range 40–66) were included in the present study. The DCIS median size was 7 mm (range 5–20 mm); six tumors were defined as grade-3, three as grade-2 and one was grade-1. Four patients had estrogen receptor-positive tumors by immunohistochemistry and six were defined as human epidermal growth factor 2 (HER)-2 positive by immunohistochemistry and FISH analysis. A written informed consent signed by each patient authorized the use of material left over from the diagnosis for research purposes. Correlations with clinical data were investigated in publicly available datasets and in a small case series from our Institution.

*TGM2* expression in stromal tissue adjacent to normal or tumor breast cells was evaluated in two publicly available datasets whose normalized data were downloaded from GEO with accession numbers GSE14548 [24] and GSE26910 [25]. Survival analysis for TGM2 was performed using METABRIC [26] and a combined dataset derived from publicly available gene expression data retrieved from GEO (GSE2034, GSE2990, GSE5327, and GSE11121). For the combined dataset, gene expression data were merged similarly as described in Callari *et al*. [13].

### 2.9. Immunohistochemistry

Four-µm thick formalin-fixed paraffin-embedded tissue sections were used for the immunohistochemistry (IHC) procedure.

After drying overnight at 37 °C, deparaffinization with xylene/ethanol and rehydration were performed. The sections were treated at 95 °C in citrate buffer for antigen retrieval, cooled and washed in TBS.

Slides were incubated with an anti-TGM2 protein primary antibody (CUB 7402, abcam ab2386) overnight at 4 °C using the Envision Dual Link System-HRP DAB+ (cat.# k5007, Dako Italia, Cernusco sul Naviglio, IT) for detection and counterstained with Mayer’s haematoxylin.

## 3. Results

The normal dermal fibroblast cell line NHDF was used for modeling the fibroblast component of the stroma. NHDFs have been previously characterized by FACS analysis with respect to the expression of superficial mesenchymal markers: CD90, CD105, CD166, CD73 confirming their mesenchymal phenotype. The CD90 marker identified two cell populations, 39.1% CD90^−^ cells and 60.9% CD90^+^ cells. For the others mesenchymal markers we observed: 97.2% CD105^+^ cells, 87.4% CD166^+^ cells and 100% CD73^+^.

Activation of the NHDF to CAF-like fibroblasts induced by treatment with BCCL conditioned medium, was checked by analyzing the expression of intracellular α- smooth muscle actin (α-SMA). Only SkBr3 increased two-fold the expression of α-SMA (8.77% *vs.* 18.2%). However, the activation status of NHDF cells upon treatment with BCCL-derived conditioned media is supported by our previous study [14], where we showed that a core of stromal genes and pathways reported as modulated in our *in vitro* model is shared with other tissue-derived and activated fibroblast from breast lesions, but also from lung and prostate cancers.

### 3.1. In Vitro Modeling of Tumor-Stroma Interactions

**Figure 1:**
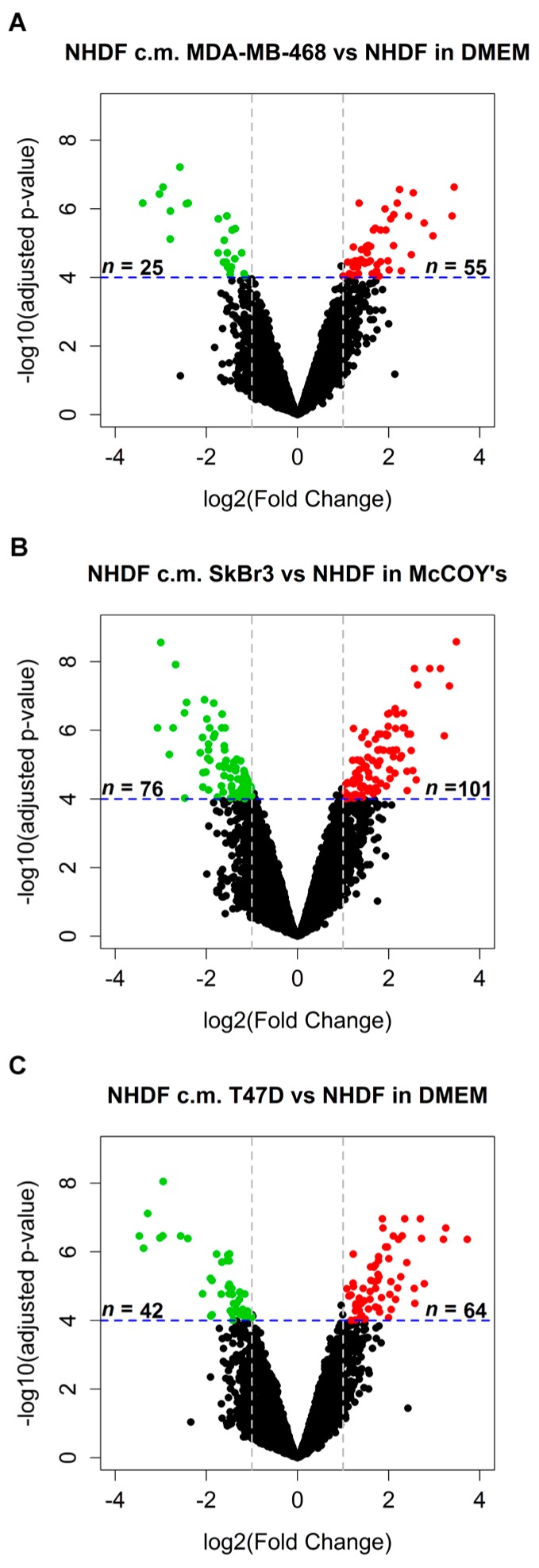
Volcano plots showing differentially expressed gene derived from Class comparison analysis NHDH treated with serum-free control medium *versus* the NHDF treated with serum-free cultured medium conditioned for 72 h by MDA-MB-468 (panel **A**: NHDF c.m. MDA-MB-231 *vs.* NHDF in DMEM), SkBr3 (panel **B**: NHDF c.m. SkBr3 *vs.* NHDF in McCOYS’s ) and T47D (panel **C**: NHDF c.m. T47D *vs.* NHDF in DMEM) cells. Dashed vertical lines delimit genes (dots) with log2 fold changes > 1, or < −1 (*x*-axis) and with high (false discovery rate FDR< 0.0001, dashed horizontal line) statistical significance (−log10 of *p*-value, *y*-axis), respectively. Black dots represent genes with fold changes and statistical significance values below the defined thresholds. Genes up-regulated above the defined cutoffs are reported as red dots, whereas down-regulated genes are reported as green dots.

BCCL-derived conditioned medium and the NF cell line NHDF were used to build an *in vitro* model mimicking stromal activation by human tumors. To such a purpose NHDF cells were treated with the conditioned media obtained from three different BCCLs under serum-free conditions, with the aim of recapitulating tumor cell secretome effects exerted in the immediate proximity of stromal cells. The three BCCLs lines were chosen to represent luminal (T47D), HER2-enriched (SkBr3) and triple negative (MDA-MB-468) cell lines. To infer gene expression alterations in the stroma induced by factors released by proximal tumor cells we compared the transcriptomic profiles of NHDF treated with conditioned medium from tumor cell lines and their respective control cells, grown in serum-free medium. Using a highly stringent significance threshold (FDR < 0.0001 and log2 fold change > 1 or < −1) we identified 80, 177 and 106 differentially expressed (DE) genes after stimulation by the triple negative MDA-MB-468 cell line, the HER2-enriched and the luminal cell line, respectively (Figure 1A–C). In all cases, upon stimulation with the tumor-derived secretome, the number of gene up-regulated in the stroma was superior to the number of down-regulated genes as can be seen from the volcano plots reported in Figure 1.

**Figure 2:**
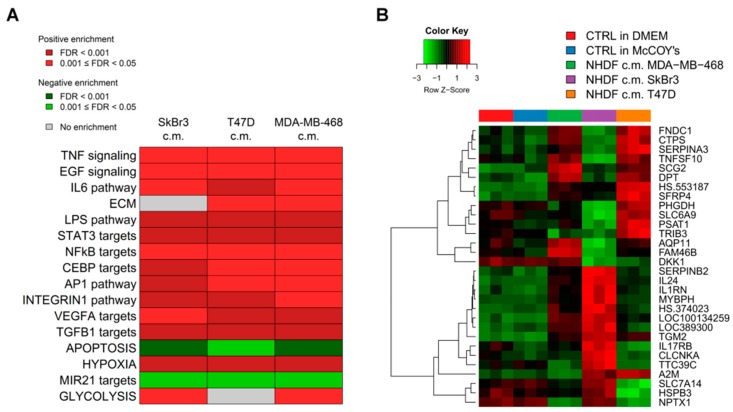
Biological interpretation of gene expression modulations in activated fibroblasts. (**A**) Selected macro biological categories found to be specifically enriched by GSEA analysis after treatment of NHDF cells with conditioned media derived from SkBr3, T47D and MDA-MB-468 cells. Positively-enriched categories are reported in red, while negatively-ones are reported in green. Color intensity reflects FDR values as shown in the inset; (**B**) Heat map of genes found to be differentially expressed (DE) across treated NHDF with an FDR < 0.0001.

To get insight into the biological processes altered upon stimulation with tumor conditioned media we performed GSEA and we identified several gene sets significantly (FDR < 0.05) positively or negatively associated to BCCL-promoted NF stimulation. To facilitate biological interpretation of the obtained results, significantly enriched gene sets were grouped into macro-categories related to specific pathways or to distinct biological functions. Results are summarized in Figure 2A.

These results show that when focusing on the macro-categories there were no differences in the biological effects exerted on the NF transcriptome as a function of the specific tumor cell line employed for stimulation (Figure 2A). However, at the single gene level there were differences in gene modulations across treated samples (Figure 2B). Moreover, given the specific type of experimental model, *i.e.* focused on paracrine effects, the stimulations were mainly observed for signaling pathways including TNF, EGF, IL-6 and TGFβ and for targets of transcription factors activated by intracellular signaling (STAT3 and NFkB). In keeping with the role of fibroblasts in the deposition of the extracellular matrix, a positive enrichment in NF conditioned cells of genes involved in ECM was also observed. Only in the case of apoptosis we observed a negative enrichment, suggesting a suppression of apoptotic pathways in the stroma in the presence of tumor foci.

**Figure 3:**
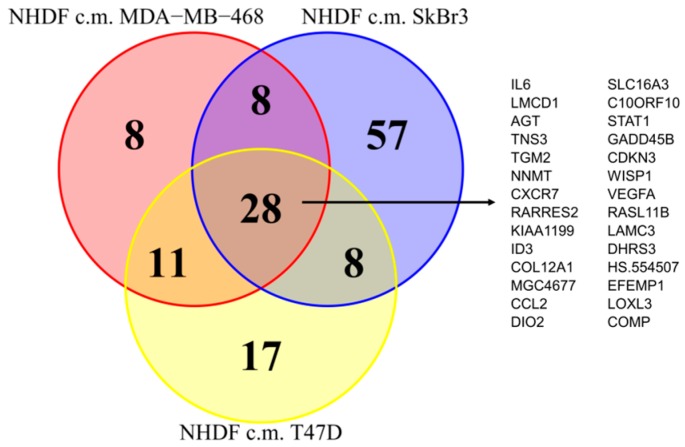
Pattern of genes up-regulated in fibroblast upon stimulation by breast cancer cells. Eulero-Venn diagrams highlighting the numbers of common and exclusive DE genes detected as up-regulated in samples derived from NHDF cells treated with serum-free medium conditioned by MDA-MB-468, by SkBr3 and by T47D breast cancer cell lines compared to their respective controls. The complete list of common up-regulated genes is also reported.

Since as reported above, the activations observed in the NF transcription program did not appear to depend on the molecular subtype of the BCCLs used for stimulation, we focused our attention on the genes commonly modulated by all three BCCLs. The lists of DE genes from each of the three comparisons (Supplementary File 1) were intersected to identify shared and private modulated genes. We identified a core of 28 genes commonly up-regulated (Figure 3) and a core set of 16 genes commonly down-regulated (Supplementary Figure S1).

Twenty-eight genes were commonly up-regulated and are listed in the right part of the Figure 3. Among the 28 up-regulated genes shared by the three comparisons we focused our attention on *TGM2*. *TGM2* gene codes for a tissue transglutaminase (TG2) that can crosslink ECM components, stabilizing the matrix and promoting cell attachment, cell motility and a general remodeling of the matrix [27]. Besides, TG2 is also considered a component of cell and tissue defense mechanisms in response to cell damage [28], making us hypothesize that it might represent an early sensor of transformation in a context where ECM plays a crucial role due to its activation in stimulated NHDF.

**Figure 4:**
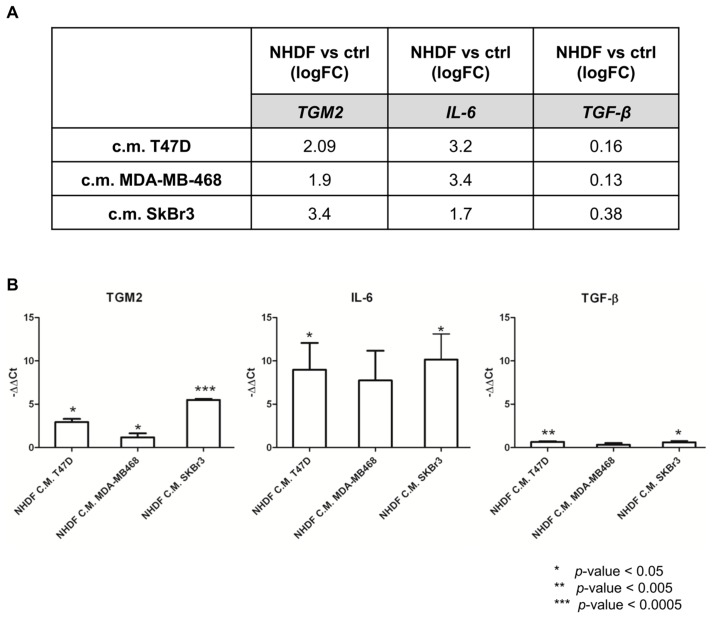
Relative expression of *TGM2*, *IL6* and *TGFB* in NHDF cells treated with breast cancer cell line-derived conditioned media. (**A**) Fold-change values (log2-tranformed) derived from class comparison analyses (treated *versus* control HNDF) of microarray data are reported in the Table; (**B**) Relative *TGM2*, *IL6* and *TGFB* expression values of HNDF treated with medium conditioned by breast cancer cell lines *versus* control media are reported. Bars represent mean –ΔΔ*C*t values (treated *C*t value—control *C*t value) of three independent biological triplicates ±SD. Statistical significance of differences between genes compared to controls were evaluated by two-tailed unpaired Student’s *t*-test.

To re-enforce the interpretation of results derived from microarray experiments, a technical validation was carried out on residual RNA derived from the same experiment for *TGM2*, *IL6* and *TGFB*. Figure 4 reports the log2-transformed fold change values derived from microarray gene expression data for three identified genes (panel A) and the −ΔΔ*C*t values obtained from qPCR measurements (panel B).

As expected, qPCR yielded larger gene expression differences compared to microarray data: however, the direction of the gene modulations observed by the two technical approaches was consistent as was the gene expression pattern. Despite *TGFB* was not included in the lists of DE genes from the microarray experiment it was included in the technical validation as the TGFβ pathway was still among the positive enriched pathways. The qPCR data confirmed the poor modulation of the gene itself.

**Figure 5:**
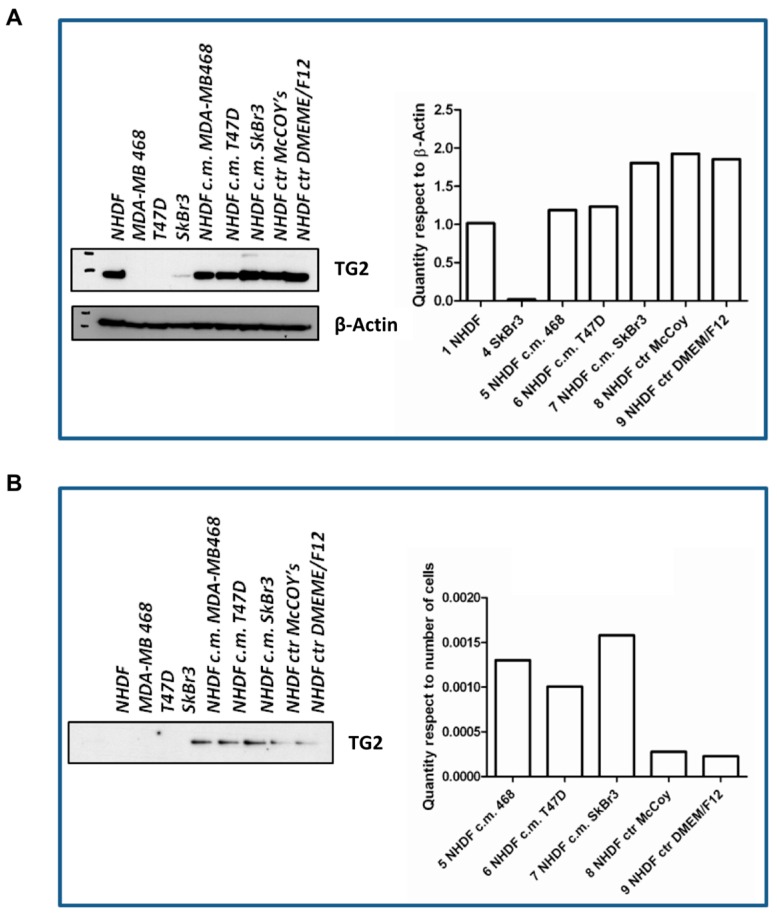
Intracellular and secreted TG2. (**A**) Western blotting results for NHDF intracellular TG2 expression upon stimulation with control medium or medium conditioned by breast cancer cell lines. The histogram reports relative quantification data by densitometry with respect to β-actin; (**B**) Western blotting results for TG2 deposited by NHDF on culture dishes upon stimulation with control or tumor cell-conditioned media. The histogram reports densitometric quantification in arbitrary units after normalization with respect to the total number of cells grown in the dish.

The *in vitro* stroma activation model was further characterized with respect to *TGM2*. To such a purpose, cells were seeded again and treated as described for the gene expression experiments using BCCL-derived conditioned media. After a 72-h treatment cell lysates were obtained for evaluation by Western blotting of TG2, the protein encoded by the *TGM2* gene (Figure 5). The TG2 protein was not expressed in our BCCLs, but was instead present in NHDF both at baseline ad after treatment with BCCL-derived conditioned media. TG2 expression was slightly up-regulated even by treatment with control culture media as well as by treatment with the conditioned media. We therefore reasoned that if, despite the up-regulation observed for the *TGM2* gene upon stimulation by tumor cells, no regulation was observed in the NHDF intracellular levels, the TG2 protein might be not retained within the cell, but immediately secreted in keeping with its role in ECM remodeling. In fact, as shown in Figure 5, neither untreated NHDF, nor BCLL line did depose any detectable amounts of TG2 on the culture dish, whereas stimulation with media conditioned by BCCL and not by control media, caused a massive increase in TG2 deposition. Finally, we excluded the presence of TG2 in the BCCL supernatants (data not shown).

### 3.2. Clinical Role of TGM2

To gain insight into the clinical role of *TGM2* we first evaluated its expression in two publicly available gene expression datasets of stromal and tumor cells laser-capture microdissected from breast cancer clinical specimens. In GSE14548 dataset, including a total of 34 stromal samples referring to stroma adjacent to normal mammary cells, DCIS or invasive ductal carcinoma (IDC) we found *TGM2* was consistently over-expressed in tumor-associated stroma with respect to stroma associated with normal mammary cells (Figure S2A). In particular, *TGM2* expression levels were increased in the stroma associated with *in situ* tumor lesions, suggesting that this gene may act as an early stromal sensor of malignant transformation, also in consideration of the slight decrease in IDC. In GSE26910 dataset, composed of 6 matched tumor and normal stroma laser-microdissected from breast tumors, we observed an up-regulation of *TGM2* in the tumor-associated stroma (Figure S2B), again suggesting that *TGM2* expression could be associated with the presence of a stimulation exerted by tumor cells similar to the one reproduced in our *in vitro* model.

**Figure 6:**
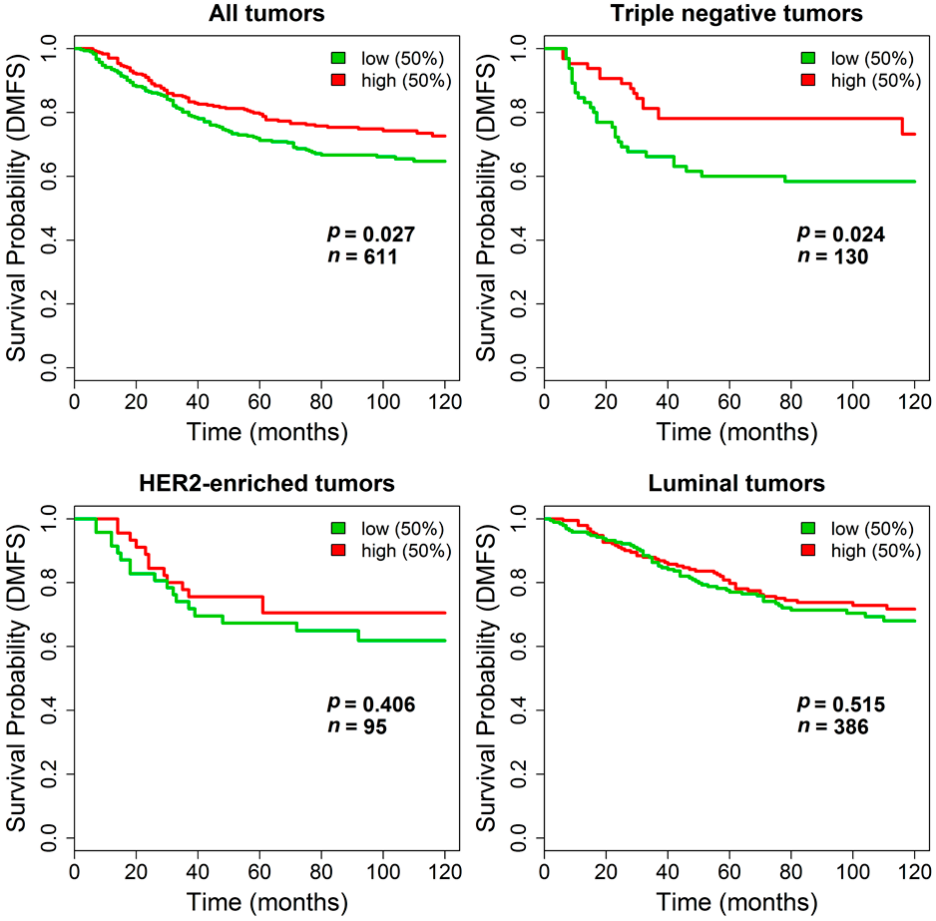
*TGM2* and distant metastasis-free survival. Kaplan-Meier analysis for the association of *TGM2* expression, stratified by median value, in tumors from patients in the combined dataset with distant metastasis-free survival (DMFS). Survival differences were evaluated by log-rank test.

We next sought to assess the association between *TGM2* expression and clinical outcome. Unfortunately, no publicly available gene expression datasets of breast cancer stromal samples with associated clinical data were available to test the prognostic value of stromal *TGM2* expression. Therefore, we could only evaluate the clinical relevance of *TGM2* in datasets derived from non-microdissected invasive tumors. When the 475 untreated lymph-node negative patients from the METABRIC dataset were stratified using the *TGM2* expression median value as cutoff, no differences in DSS were observed by survival analysis both considering all patients or by separately analyzing the patients subdivided according to their tumor molecular subtype. Kaplan-Meyer curves are reported in Supplementary Figure S3. In contrast, in the combined dataset including stage I breast cancers from untreated patients, where the absence of treatment and the information on DMFS allowed testing pure prognostic effects, stratification by *TGM2* expression revealed a statistically significant longer DMFS in patients with primary tumors expressing higher *TGM2* levels (log-rank *p =* 0.027, *n =* 611). The impact of *TGM2* expression on DMFS was even stronger in the subset of triple negative tumors (log-rank *p =* 0.024, *n =* 130) whereas it was negligible in patients bearing HER2-enriched (log-rank *p =* 0.41, *n =* 95) or luminal tumors (log-rank *p* = 0.51, *n* = 386). Kaplan-Meier curves are reported in Figure 6.

As already stated all the above data refer to non-microdissected tumors and, despite the increased percentage of tumor cells with respect to stromal compartment in the analyzed samples, the possibility that the contribution of stroma may impact on the data cannot be excluded.

Considering our hypothesis that TG2 might represent an early sensor of tumor-induced modification in the stroma, we evaluated TG2 expression at protein level by IHC separately in stroma and in the tumor compartments of 5 patients diagnosed with DCIS and who later developed in invasive tumor matched with 5 women of similar age and diagnosed with DCIS lesions of comparable grade and size, albeit free of invasive relapse for a similar follow up duration.

An example of TG2 staining in the stromal and epithelial compartments is shown in Supplementary Figure S4. Remarkably, all sections showed positivity for endothelial cell. Forty percent of sections showed a moderate-to-strong cytoplasmic positivity in neoplastic cells and a moderate-to-strong positivity of fibroblast cells in peritumoral stroma. In 20% of the cases, neoplastic cells of DCIS were negative for TG2, but peritumoral fibroblast showed cytoplasmic positivity.

Among women known to have progressed to an invasive lesion after DCIS diagnosis, the TG2 expression in the tumor cells was trendily (*p =* 0.077, two-tailed Student’s *t* test) associated with a longer DFS (77 ± 15.5 months) compared to women with TG2 negative DCIS (46.3 ± 11 months). No differences were instead observed stratifying the women by stromal expression of TG2. Similarly, when looking at patients defined as controls, who represent a similar subset of women with comparable DCIS lesions, but who have not developed an invasive lesion at a similar observation time, stromal and tumoral did not associate as expected with any differences in follow-up time. This suggests that a high tumoral rather than stromal TG2 expression might play a role in predicting subsequent invasive evolution. In fact, when considering only women whose tumors did in fact undergo a progression towards the invasive phenotype, all women with a late progression after 5 years (3/3) were positive in their tumor cells, whereas those with an early progression (<5 years) had TG2 negative *in situ* lesions (2/2). No such association could be observed in the stromal compartment. Importantly, grading was not associated with early or late progression to invasive phenotype.

## 4. Discussion

In the last two decades, the role of the microenvironment as one of the crucial hallmarks in cancer has been increasingly recognized, while a raising amount of evidences has been accumulated on the actual participation of normal cells in the acquisition of cancer hallmarks [29].

In the mammary gland the role of the microenvironment is important both in the development of the organ and in its neoplastic transformation [30]. Fibroblasts in particular, are known to play a major role on the stage of the microenvironment [31], and we are understanding that not all fibroblasts act in the same way [32]. The heterogeneous nature of breast cancer both at molecular and clinical level, although extensively unraveled [33], still poses more questions than answers since robust biomarkers of proven clinical utility are still lacking. In such a context the characterization of the tumor microenvironment through gene expression profiling proved to inform on the clinical outcome, albeit no biomarkers of clinical usefulness have been so far developed (reviewed in [7]).

So far, to the best of our knowledge, the search for biomarkers of stromal activation with translational purposes has rarely followed an approach based on the generation of *in vitro* models. We therefore tried to address such a gap by developing a model for breast cancer. The approach is very similar to the one already previously used in our laboratory for modeling tumor cell activation by stromal cells, and which proved to have a translational value as it generated a molecular subtype-specific gene signature associated with increased risk of developing distant metastases in women with early breast tumors (manuscript submitted).

The biological reliability of the present model is supported by the satisfactory overlap between both the genes and the biological categories identified as modulated in our model and activated fibroblasts derived from similar and different contexts (including both *ex vivo* and *in vitro* samples) such as lung and prostate tumors [14]. This finding gives an indirect support to the reliability of the candidate genes obtained by our approach.

When specific genes involved in fibroblasts activation were examined, the biological categories related to intracellular signaling activation and the genes herein were among the strongest candidates for becoming stromal activation biomarkers. They were however, excluded from our screen for candidate biomarkers, since signaling activation (although being definitely expected given the paracrine type of stimulation) represents a category at the crossroad of many pathways. In contrast, the increased expression of the *TGM2* gene represented an interesting possible candidate. This gene encodes for a tissue transglutaminase 2, a multifunctional protein with many enzymatic activities spanning from transglutaminase, GTPase/ATPase, protein disulfide isomerase, and protein kinase, but also entangled with non-enzymatic functions due to its interaction with multiple cellular proteins. Finally, TG2, the protein encoded by *TGM2* is involved in modulation and deposition of extracellular matrix and is up-regulated in inflammation and wound [28,34].

Since *TGM2* with its multifunctional role could represent an interesting candidate, after confirming the up-regulation of the gene with an orthogonal assay, we evaluated whether in our activated fibroblasts its up-modulation affected the intracellular or the secreted encoded protein or both, and confirmed that treatment with tumor cell-derived conditioned medium was responsible for an increase in the secreted protein. In fact, we could observe a massive increase in TG2 protein levels among the protein deposed on the cell culture dishes and corresponding to the ECM released by fibroblasts. This latter finding further increased our interest in this protein since it represents a tumor-stimulated stromal target directly affecting the ECM and with a potential, thanks to its enzymatic activity, to modify the EMT texture eventually affecting tumor cell migration.

At difference with the data reported by Park *et al*. [35] and Tchou *et al*. [36], we did not observe molecular subtype–specific gene modulations in our activated fibroblasts. This could be an indication of the fact that our model captures the very early alterations induced in fibroblast in response to the activating tumor secretome, whereas modulations observed in clinical samples are the result of a long-time crosstalk between fibroblasts and tumor cells.

Being convinced, based on our *in vitro* experiments, of the potential of *TGM2* as candidate biomarker acting as an early sensor of stromal activation, we preceded with evaluating its clinical role. This was initially done in two public dataset of invasive tumors, an approach presenting limitations that we were aware of. The first limitation relates to the fact that we had identified *TGM2* as a potential early marker, but were instead looking at it role in invasive tumors, which did not at all represent the situation of a naïve normal stroma undergoing a stimulation derived from the very early steps of malignant transformation. The second limitation relates to the nature of the clinical sample itself, which is not representative of the stroma, but is a non-pure, but still tumor-cell enriched rather than stroma-cell enriched tissue specimen. In fact, this evaluation resulted in a rather unexpected result with *TGM2* expression being associated with a longer DMFS in the general population, a finding which, was probably driven by the minority of basal-like tumors where the ‘protective’ effect of a high *TGM2* expression seemed to be stronger.

We therefore turned our attention to the clinical setting of DCIS representing a non-obligate precursor of invasive tumors and where, as explained in the introduction, the need for a biomarker of invasive evolution is very strong. This time we could overcome the limitation linked to mixed samples as by using IHC we separately evaluated the stromal and tumor cells expression of TG2. In this context, the outcome considered for evaluating the role of TG2 expression was the time to invasive evolution after the diagnosis and the surgical removal of an *in situ* lesion. The sample size was quite limiting, but we still could confirm a protective role for the tumor cell-associated TGM2 (albeit for the protein and not the gene) similar to the one reported in infiltrating tumors. Conversely, stromal TG2 expression was not associated with an earlier progression to invasive disease, a finding that could appear to be in contrast with our hypothesis, although a possible explanation can be given as explained below.

Where the limited sample size does not help in supporting strong and definitive conclusions, the obtained results might however suggest that the role of stromal TG2 should be better evaluated in earlier precursor lesions such as atypic ductal hyperplasia, atypical apocrine hyperplasia, microglandular adenosis [37]. DCIS, besides representing a real clinical dilemma, is an already established tumor that even retains its molecular subtype from *in situ* to invasive disease. This is supported by pathological considerations, but also by gene expression studies [24]. In fact, in their seminal paper Ma *et al.* [24] profiled for gene expression 14 patient-matched samples from normal epithelium, normal stroma and tumor including both the invasive and the *in situ* lesions. In the epithelium gene expression changes occurred only in the transition from normal cells to DCIS, and similarly in the stroma, the very great majority of gene expression modulations were again occurring in the transition from normal tissue to DCIS. This would therefore provide a possible explanation for the failed validation of the stromal *TGM2* role as early sensor of transition to an invasive phenotype, since DCIS would already share the same traits of the invasive lesion and would not represent the ideal model for early stromal activation. On the other hand, the outcome that we have considered (invasive tumor occurring before or after 5 years) for our evaluations of the clinical role of TG2 might not be the best surrogate for a true early stromal reaction.

A literature study [38] reports data on tumor and stromal TG2 expression in 44 patients with *in situ* breast lesions in combination with HJURP and HIF. In addition, in this larger case series no correlation was found between neither tumor nor stromal TG2 expression and relapse (both *in situ* and invasive) occurring within 12 months from surgery in the ipsilateral breast. However, similarly to our study the authors report higher TG2 expression in tumor cells in non-relapsed compared to relapsed patients.

Therefore, we feel that *TGM2* with its potential role as a modifier of the ECM texture represents an extremely interesting biomarker, but that the clinical validation should probably address earlier pre-neoplastic lesions rather than DCIS. Accordingly, *TGM2* might represent a diagnostic rather than prognostic biomarker. Despite the failed clinical validation, these results are still biologically relevant as supported by the above mentioned overlapping of our up-regulated genes with other genes obtained from clinically derived and from *in vitro* models of cancer activated fibroblasts, and would therefore deserve a validation in a different clinical context.

## Supplementary Materials

The following are available online at http://www.mdpi.com/2076-3905/5/2/10/s1.

## References

[1] Egeblad M., Nakasone E.S., Werb Z. (2010). Tumors as organs: Complex tissues that interface with the entire organism. Dev. Cell.

[2] Pietras K., Ostman A. (2010). Hallmarks of cancer: Interactions with the tumor stroma. Exp. Cell Res..

[3] Polyak K., Haviv I., Campbell I.G. (2009). Co-evolution of tumor cells and their microenvironment. Trends Genet..

[4] Hanahan D., Coussens L.M. (2012). Accessories to the crime: Functions of cells recruited to the tumor microenvironment. Cancer Cell.

[5] Quail D.F., Joyce J.A. (2013). Microenvironmental regulation of tumor progression and metastasis. Nat. Med..

[6] Erickson A.C., Barcellos-Hoff M.H. (2003). The not-so innocent bystander: The microenvironment as a therapeutic target in cancer. Expert Opin. Ther. Targets.

[7] Giussani M., Merlino G., Cappelletti V., Tagliabue E., Daidone M.G. (2015). Tumor-extracellular matrix interactions: Identification of tools associated with breast cancer progression. Semin Cancer Biol..

[8] Conklin M.W., Keely P.J. (2012). Why the stroma matters in breast cancer: Insights into breast cancer patient outcomes through the examination of stromal biomarkers. Cell Adh. Migr..

[9] Zou W. (2006). Regulatory T cells, tumour immunity and immunotherapy. Nat. Rev. Immunol..

[10] Pollard J.W. (2004). Tumour-educated macrophages promote tumour progression and metastasis. Nat. Rev. Cancer.

[11] Dushyanthen S., Beavis P.A., Savas P., Teo Z.L., Zhou C., Mansour M., Darcy P.K., Loi S. (2015). Relevance of tumor-infiltrating lymphocytes in breast cancer. BMC Med..

[12] Karn T., Pusztai L., Rody A., Holtrich U., Becker S. (2015). The Influence of Host Factors on the Prognosis of Breast Cancer: Stroma and Immune Cell Components as Cancer Biomarkers. Curr. Cancer Drug Targets.

[13] Callari M., Cappelletti V., D’Aiuto F., Musella V., Lembo A., Petel F., Karn T., Iwamoto T., Provero P., Daidone M.G. (2016). Subtype-Specific Metagene-Based Prediction of Outcome after Neoadjuvant and Adjuvant Treatment in Breast Cancer. Clin. Cancer Res..

[14] Gandellini P., Andriani F., Merlino G., D’Aiuto F., Roz L., Callari M. (2015). Complexity in the tumour microenvironment: Cancer associated fibroblast gene expression patterns identify both common and unique features of tumour-stroma crosstalk across cancer types. Semin Cancer Biol..

[15] Qiao A., Gu F., Guo X., Zhang X., Fu L. (2016). Breast cancer-associated fibroblasts: Their roles in tumor initiation, progression and clinical applications. Front. Med..

[16] Benson J.R., Wishart G.C. (2013). Predictors of recurrence for ductal carcinoma *in situ* after breast-*conserving* surgery. Lancet Oncol..

[17] Silverstein M.J., Poller D.N., Waisman J.R., Colburn W.J., Barth A., Gierson E.D., Lewinsky B., Gamagami P., Slamon D.J. (1995). Prognostic classification of breast ductal carcinoma-*in situ*. Lancet.

[18] Rudloff U., Jacks L.M., Goldberg J.I., Wynveen C.A., Brogi E., Patil S., Van Zee K.J. (2010). Nomogram for predicting the risk of local recurrence after breast-conserving surgery for ductal carcinoma *in situ*. J. Clin. Oncol..

[19] Livak K.J., Schmittgen T.D. (2001). Analysis of relative gene expression data using real-time quantitative PCR and the 2(−ΔΔ*C*t) Method. Methods.

[20] Du P., Kibbe W.A., Lin S.M. (2008). Lumi: A pipeline for processing Illumina microarray. Bioinformatics.

[21] Barrett T., Troup D.B., Wilhite S.E., Ledoux P., Evangelista C., Kim I.F., Tomashevsky M., Marshall K.A., Phillippy K.H., Sherman P.M. (2011). NCBI GEO: Archive for functional genomics data sets—10 years on. Nucleic Acids Res..

[22] Smyth G.K. (2004). Linear models and empirical bayes methods for assessing differential expression in microarray experiments. Stat. Appl. Genet. Mol. Biol..

[23] Subramanian A., Tamayo P., Mootha V.K., Mukherjee S., Ebert B.L., Gillette M.A., Paulovich A., Pomeroy S.L., Golub T.R., Lande E.S. (2005). Gene set enrichment analysis: A knowledge-based approach for interpreting genome-wide expression profiles. Proc. Natl. Acad. Sci. USA.

[24] Ma X.J., Dahiya S., Richardson E., Erlander M., Sgroi D.C. (2009). Gene expression profiling of the tumor microenvironment during breast cancer progression. Breast Cancer Res..

[25] Planche A., Bacac M., Provero P., Fusco C., Delorenzi M., Stehle J.C., Stamenkovic I. (2011). Identification of prognostic molecular features in the reactive stroma of human breast and prostate cancer. PLoS ONE.

[26] Dvinge H., Git A., Graf S., Salmon-Divon M., Curtis C., Sottoriva A., Zhao Y., Hirst M., Armisen J., Miska E.A. (2013). The shaping and functional consequences of the microRNA landscape in breast cancer. Nature.

[27] Nurminskaya M.V., Belkin A.M. (2012). Cellular functions of tissue transglutaminase. Int. Rev. Cell Mol. Biol..

[28] Agnihotri N., Kumar S., Mehta K. (2013). Tissue transglutaminase as a central mediator in inflammation-induced progression of breast cancer. Breast Cancer Res..

[29] Hanahan D., Weinberg R.A. (2011). Hallmarks of Cancer: The Next Generation. Cell.

[30] Polyak K., Kalluri R. (2010). The role of the microenvironment in mammary gland development and cancer. Cold Spring Harb. Perspect Biol..

[31] Kalluri R., Zeisberg M. (2006). Fibroblasts in cancer. Nat. Rev. Cancer.

[32] Sugimoto H., Mundel T.M., Kieran M.W., Kalluri R. (2006). Identification of fibroblast heterogeneity in the tumor microenvironment. Cancer Biol. Ther..

[33] Prat A., Ellis M.J., Perou C.M. (2011). Practical implications of gene-expression-based assays for breast oncologists. Nat. Rev. Clin. Oncol..

[34] Mehta K., Kumar A., Kim H.I. (2010). Transglutaminase 2: A multi-tasking protein in the complex circuitry of inflammation and cancer. Biochem. Pharmacol..

[35] Park S.Y., Kim H.M., Koo J.S. (2015). Differential expression of cancer-associated fibroblast-related proteins according to molecular subtype and stromal histology in breast cancer. Breast Cancer Res. Treat..

[36] Tchou J., Kossenkov A.V., Chang L., Satija C., Herlyn M., Showe L.C., Puré E. (2012). Human breast cancer associated fibroblasts exhibit subtype specific gene expression profiles. BMC Med. Genomics.

[37] Lopez-Garcia M.A., Geyer F.C., Lacroix-Triki M., Marchiò C., Reis-Filho J.S. (2010). Breast Cancer precursors revisitated: Molecular features and progression pathways. Histopathology.

[38] Bravaccini S., Tumedei M.M., Scarpi E., Zoli W., Rengucci C., Serra L., Curcio A., Buggi F., Folli S., Rocca A. (2014). New biomarkers to predict the evolution of *in situ* breast cancers. Biomed. Res. Int..

