# Supplementary material for: Stromal Activation by Tumor Cells: An in Vitro Study in Breast Cancer
Source: Microarrays (Basel). 2016 May 18;5(2):10. doi: 10.3390/microarrays5020010 (PMC5003486; doi:10.3390/microarrays5020010)
Supplement: Supplementary file 1 [file microarrays-05-00010-s001.pdf]

Supplementary Materials: Stromal Activation by Tumor Cells: An *in Vitro* Study in Breast Cancer

Giuseppe Merlino [1], Patrizia Miodini [1], Biagio Paolini [2], Maria Luisa Carcangiu [2], Massimiliano Gennaro [3], Matteo Dugo [4], Maria Grazia Daidone [1] and Vera Cappelletti [1,*]

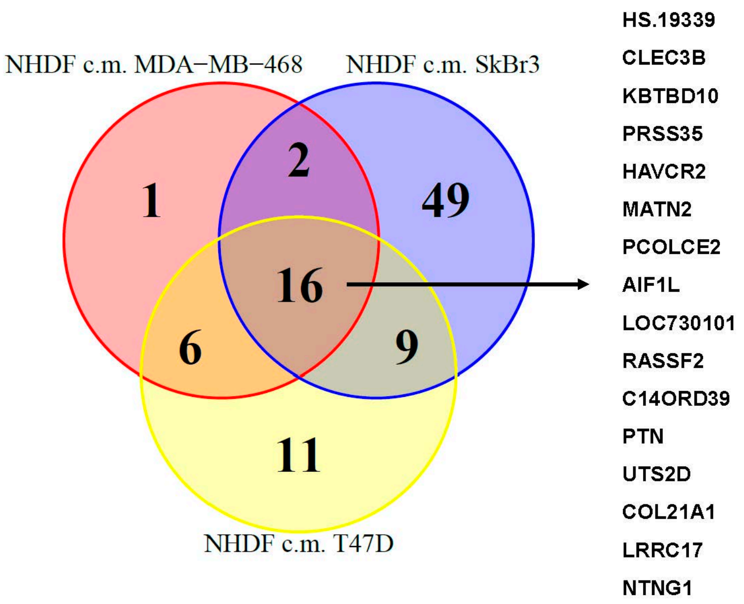

Figure S1. Venn diagram reporting common and private down-regulated genes in the class comparisons. Eulero-Venn diagram highlighting the numbers of differentially expressed (FDR < 0.0001) common and exclusive genes detected as down-regulated in samples derived from NHDF cells treated with serum-free medium conditioned by MDA-MB- 468, by SkBr3 and by T47D breast cancer cell lines compared to their controls. The complete list of common down-regulated genes is also reported.

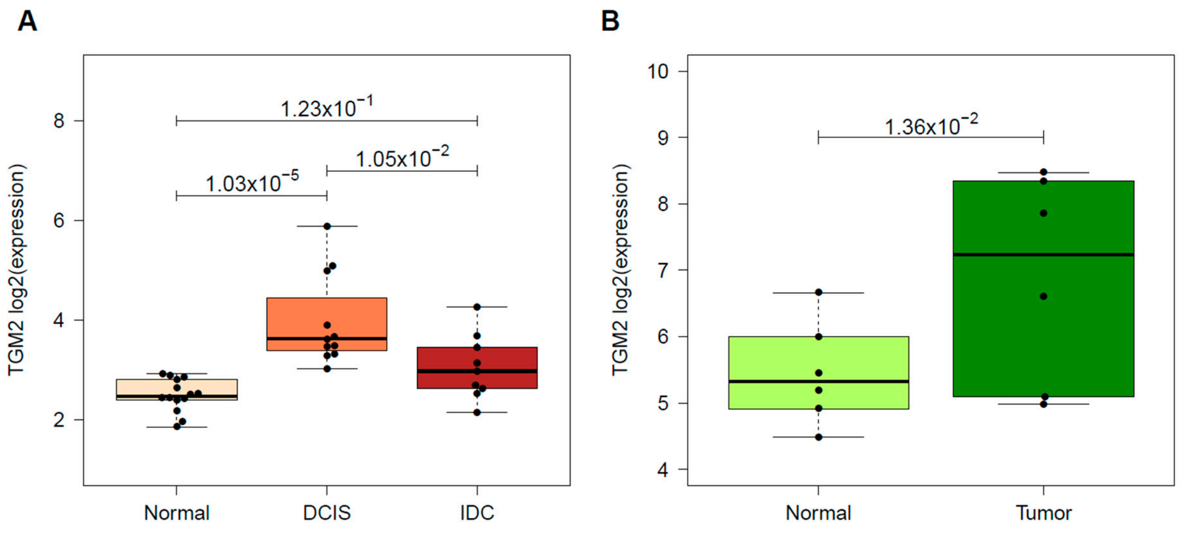

Figure S2. Expression of stromal TGM2 in literature datasets. (**A**) Boxplots of expression pattern of *TGM2* as derived from microarray data (Ma *et al.*, ref [26]) in stromal cells adjacent to normal mammary cells, to DCIS and to invasive (IDC) lesions. Statistical significance was tested by ANOVA followed by Tukey's post-*hoc* test; (**B**) Boxplots of expression pattern of *TGM2* as derived from microarray data (Planche *et al.*, ref [27]) in stromal cells adjacent to normal mammary cells and to tumor lesions. Statistical significance was assessed by two- tailed paired Student's *t*-test.

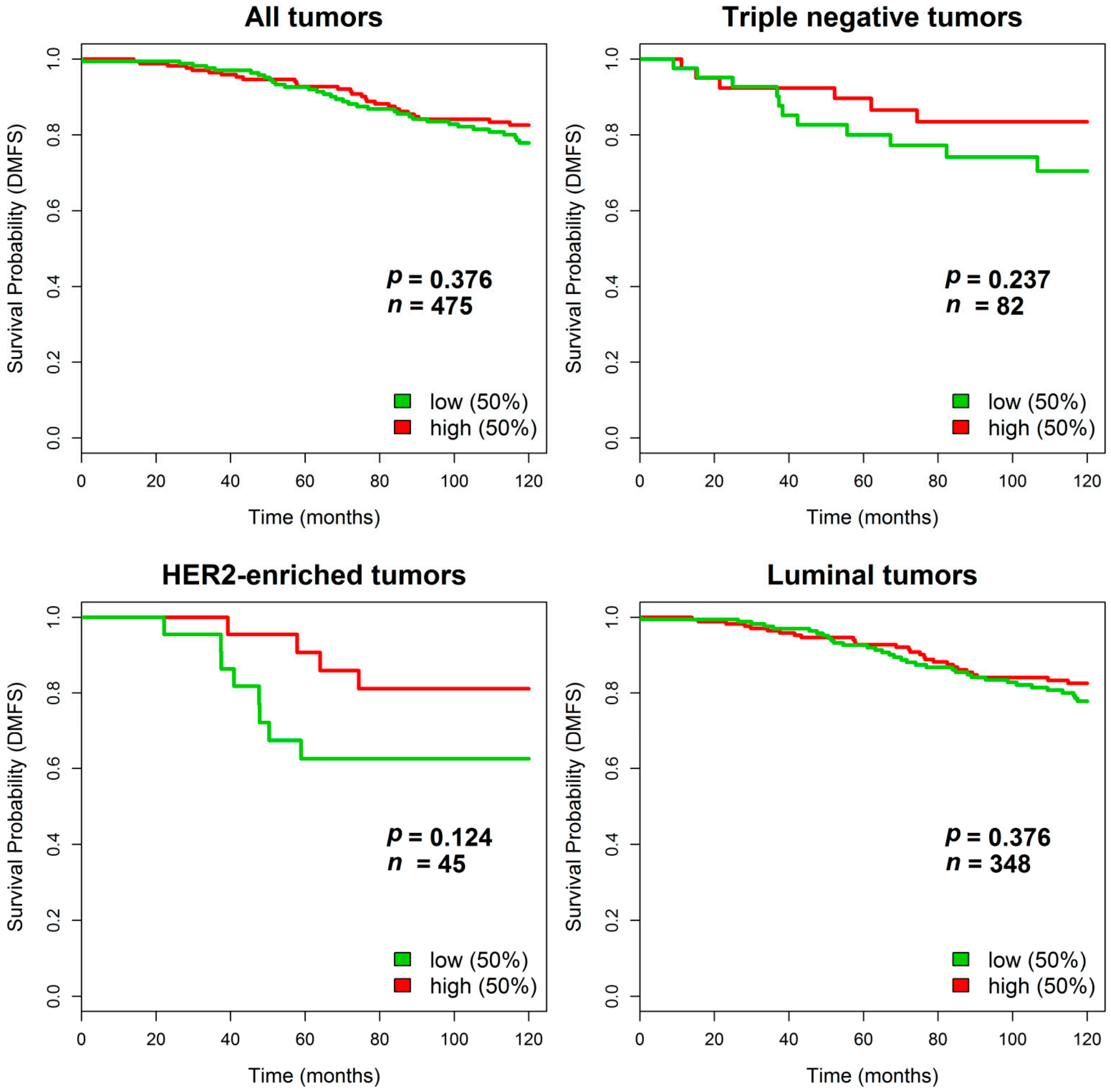

Figure S3. Kaplan-Meier analysis for the association of TGM2 expression stratified by median value in tumors from patients from the METABRIC dataset with disease-specific overall survival (DSS). Survival differences were evaluated by log-rank test.

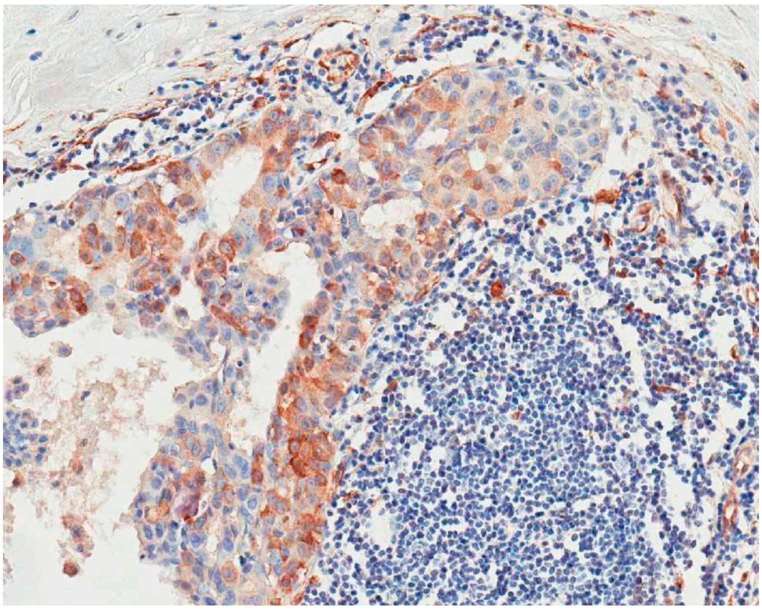

(**A**)

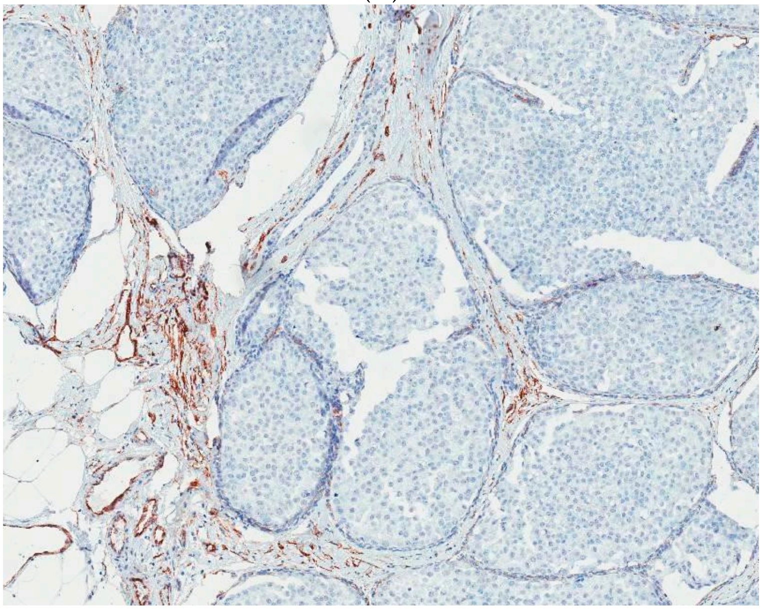

(**B**)

Figure S4. TG2 staining in clinical sections from patients with DCIS. (**A**) Strong staining in tumor cells; (**B**) Positive staining for TG2 in stromal cells. Magnification 20×.
